# Body Mass Index and Gestational Weight Gain During Pregnancy and Foetomaternal Outcomes: A Cohort Study at a Tertiary Health Facility in Lagos, Nigeria

**DOI:** 10.7759/cureus.93684

**Published:** 2025-10-01

**Authors:** Oluwaseun E Familusi, Tersur T Saalu, Simeon Jiekhename, Lucky E Tietie, Charity O Maduagu, Olufunso J Naiyeju, Chidiebere A Agbo, Aanuoluwa T Edema, Margaret A Reuben, Ochuwa A Babah

**Affiliations:** 1 Obstetrics and Gynaecology, Lagos University Teaching Hospital, Lagos, NGA

**Keywords:** body mass index, foetal outcome, gestational weight gain, maternal outcome, nigeria, pregnancy

## Abstract

Introduction: Both body mass index (BMI) and gestational weight gain (GWG) may play a significant role in predicting events that could impact maternal and foetal health.

Objectives: We determined the association between BMI and GWG versus foetomaternal outcomes and their accuracy in predicting adverse pregnancy outcomes.

Methodology: A cohort study of 231 pregnant women with a singleton foetus at ≤20 weeks of gestation, who received antenatal care and delivery at Lagos University Teaching Hospital, Lagos, Nigeria, over three years between August 1, 2019, and July 31, 2022 was conducted. Data was extracted from case notes. The exposures were BMI and GWG, while the outcomes were the incidence of foetomaternal complications. Associations between categorical variables were determined using the chi-square test, and the receiver operating characteristic curve was used to determine accuracy.

Results: The mean age of participants was 32.7 ± 5.4 years. BMI was found to be associated with an increased incidence of maternal mortality (p = 0.006), foetal macrosomia (defined as birth weight >4 kg, p = 0.001), and stillbirths (p = 0.008). Conversely, GWG showed no significant association with any of the outcome measures (p > 0.05). Both BMI, with an area under the curve (AUC) of 0.547 (95% CI: 0.472-0.723), and GWG, with an AUC of 0.561 (95% CI: 0.487-0.635), were not meaningful predictors of adverse pregnancy outcomes.

Conclusion: BMI and GWG are poor predictors of adverse pregnancy outcomes. However, underweight and obese pregnant women should be considered high-risk because of a higher incidence of stillbirth, foetal macrosomia, and maternal death.

## Introduction

Body mass index (BMI) serves as a valuable tool in assessing an individual's nutritional status [1]. BMI is calculated using both the weight and height [1]. BMI can be used to categorise people into underweight, normal weight, overweight, and obese [2]. According to the Centers for Disease Control and Prevention (CDC), a normal weight refers to a BMI of 18.5-24.9kg/m^2^, while underweight, overweight, and obesity refer to a BMI of <18.5 kg/m2, 25-29.9 kg/m^2^, and ≥ 30 kg/m^2^, respectively [2]. There is an increase in the prevalence of both overweight and obesity among women of reproductive age [3]. Obesity has been reported to be a global health challenge [4]. Worldwide, about 20-36% of women of reproductive age are obese [5]. Similarly, an increase in the prevalence of obesity has been noted among pregnant women [6]. It is important to determine the BMI status of every pregnant woman at the first antenatal clinic so that they can be counselled about the risks of their BMI status, especially if they are either underweight, overweight, or obese [7]. Obesity is associated with adverse pregnancy outcomes like hypertensive disorder of pregnancy, gestational diabetes, increased risk of caesarean section, postpartum haemorrhage, venous thromboembolism, and maternal mortality [8,9]. The prevalence of underweight women in low- to middle-income countries is higher than that of the developed countries [10]. Underweight pregnant women may experience preterm delivery, increased risk of intrauterine growth restriction and foetal morbidity, anaemia, and maternal infection [11-14].

Gestational weight gain (GWG) can affect the health status of pregnant women and their babies [15]. The expected weight gain in pregnancy is dependent on pre-pregnancy BMI status [15]. According to the updated GWG guideline, the optimal weight gain for underweight, overweight, and obese individuals is 12.5-18 kg, 7-11.5 kg, and 5-9 kg, respectively [16]. An optimal weight gain during pregnancy will help achieve a favourable pregnancy outcome [15]. Maternal age, race, parity, and pre-pregnancy BMI have been identified as determinants of the amount of weight gained during pregnancy [17]. Excessive and insufficient GWG has been associated with adverse pregnancy outcomes such as small for gestational age, large for gestational age, macrosomia, caesarean delivery, gestational diabetes mellitus, hypertensive disorder of pregnancy, childhood obesity, and postpartum weight retention [18-21].

This study determined the association between BMI and GWG versus foetomaternal outcomes, and the accuracy of BMI and GWG alone in predicting adverse pregnancy outcomes.

## Materials and methods

Study design

This was a retrospective cohort study. This study design was adopted to allow a clearer understanding of the temporality of the association between the predictors and outcomes studied.

Study location

This study was conducted at the Lagos University Teaching Hospital (LUTH), Lagos. LUTH is the largest tertiary health facility in Lagos state, located in the South-western geopolitical zone of Nigeria. It acts mainly as a referral centre for other publicly owned and private health facilities within the state and other neighbouring states. It has multidisciplinary teams and is equipped to manage complicated obstetric cases. It registers on average 120 - 150 new pregnant women for antenatal care monthly, with about 100 deliveries monthly.

Study population

A total of 231 pregnant women with a singleton foetus at ≤20 weeks of gestation, who had antenatal care and delivery at the facility over three years between August 1, 2019, and July 31, 2022, were included in the study.

Inclusion criteria

The study included women with singleton pregnancies, those who received their antenatal care at LUTH (booked patients), and women who registered for antenatal care in the first trimester of pregnancy.

Exclusion criteria

Excluded from this study were women with multiple pregnancies, women who booked late after 13 weeks, and women who did not receive antenatal care in LUTH.

Sample size calculation

Using Cleveland Clinic’s cohort study calculator for sample size calculation (https://riskcalc.org/samplesize/), a sample size of 118 (59 per group) was calculated to be adequate to detect a statistically significant difference in the incidence of any maternal complication occurring in obese versus non-obese pregnant women at 80% power and 95% confidence level, based on findings from a previous study in Nigerian which found incidence of maternal complications to be 25.9% in obese pregnant women versus 6.2% in the non-obese pregnant women [22], considering 10% attrition rate. However, we included in the sample all 231 eligible pregnant women with traceable records who had antenatal care and delivery at the facility during the study period. 

Data collection

Data were extracted from the case notes, including sociodemographic information such as age, parity, and booking status (indicating whether antenatal care was received at the facility). The obstetric history documented included gestational age, presence of comorbidities, and mode of delivery. Additionally, foetal complications such as preterm birth, stillbirths, foetal macrosomia, and neonatal unit admissions were noted, along with any maternal complications and cases of maternal death. Furthermore, the individuals' height and initial weight measurements were recorded.

Outcome measures

Exposures were BMI categorized as underweight (<18.5kg/m^2^), normal (18.5-24.9kg/m^2^), overweight (25.0-29.9kg/m^2^) and obese (≥30.0kg/m^2^); and GWG categorized as inadequate (<11 kg), normal (11-16 kg) and excessive (>16 kg). The primary outcome was the incidence of any maternal complications. The maternal complications comprised obstetric complications like obstructed labour, perineal tears, postpartum haemorrhage, cervical lacerations, failure to progress, cephalopelvic disproportion and medical conditions like preeclampsia, gestational hypertension and gestational diabetes mellitus. Maternal complication was categorised as a dichotomous variable - Yes or No, if the mother had any complication or if she did not suffer any complication during pregnancy and delivery, respectively. The secondary outcomes were the incidences of maternal death, preterm birth, stillbirths, foetal macrosomia, and neonatal unit admission.

Data analysis

The data were analysed using IBM SPSS Statistics for Windows, Version 20 (Released 2011; IBM Corp., Armonk, New York, United States). Descriptive statistics were done. Continuous variables that were not normally distributed like BMI, maternal weight at booking, maternal weight at delivery and GWG were presented as median and interquartile range. Categorical variables like age group, parity, pre-existing comorbidities, pregnancy complications and mode of delivery were presented as frequency with percentage or as proportions. Associations between BMI and pregnancy outcomes were determined using the chi-square test; Fisher's exact test was used where the expected value in more than 20% of the cells was less than 5. The association between GWG and pregnancy outcomes was determined using Fisher's exact test. The receiver operator characteristic curve was used to determine the accuracy of BMI and GWG for predicting adverse pregnancy outcomes. There was no imputation for missing data. Statistical significance was set at p-value < 0.05, considering a two-tailed hypothesis.

Ethics

Ethical approval was obtained from the Lagos University Teaching Hospital Health Research Ethics Committee (ADM/DSCST/HREC/APP/5423) before the commencement of this study. Data confidentiality was upheld, and the data was stored on a computer protected by a password.

## Results

The mean age of the participants was 32.7 ± 5.4 years, with a median parity of 2 (IQR: 1-3). Of the women, 55 (23.8%) experienced complications during pregnancy. Table 1 presents details of the sociodemographic characteristics of the participants.

**Table 1:** Sociodemographic and clinical profile of study participants (n = 231)

Sociodemographic and clinical characteristics	Frequency (n = 231)	Percentage (%)
Age group (years)		
21 – 29	65	28.1
30 – 39	139	60.2
40 – 49	26	11.3
50 and above	1	0.4
Parity		
0 – 1	87	37.7
2 – 4	133	57.6
≥5	11	4,8
Pre-existing comorbidities		
Yes	43	18.6
No	188	81.4
Pregnancy complications		
Yes	55	23.8
No	176	76.2
Mode of delivery		
Vaginal delivery	115	49.8
Caesarean section	115	49.8

The median BMI of the participants was 27.2 (IQR: 23.4 - 30.8) kg/m², and a median GWG of 7.5 (IQR: 5.0 - 10.8) kg. Table 2 presents the details of the clinical profile of the participants.

**Table 2:** Clinical profile of study participants (n = 231)

Clinical characteristics	Median	Interquartile range
Body mass index at booking (kg/m^2^)	27.2	23.4 – 30.8
Maternal weight at booking (kg)	70	60.0 – 82.0
Maternal weight at delivery (kg)	80	68.0 – 91.0
Gestational weight gain (kg)	7.5	5.0 – 10.8

Table 3 presents a summary of the association between BMI and pregnancy outcomes. The incidence of caesarean section was higher in women with abnormal BMI (underweight, overweight, and obese) compared to those with normal BMI, p = 0.013. Pregnant women who were underweight or obesity had a significantly higher incidence of maternal death compared those who had normal BMI or were overweight, 1 (14.3%) in underweight, 2 (2.9%) in obese versus none in normal and overweight categories, p = 0.006; foetal macrosomia (birth weight >4 kg) 9 (11.7%) in overweight and 8 (11.4%) in obese versus none in normal and underweight categories, p = 0.001; stillbirths 1 (14.3%) in underweight, 3 (4.3%) in obese versus none in normal and overweight categories, p = 0.008.

**Table 3:** Association between body mass index and pregnancy outcomes (n = 231) *Chi-square test; # Fisher's exact test. One obese pregnant woman did not have her mode of delivery recorded. One underweight and three obese pregnant women did not have a record for the APGAR score. SVD: Spontaneous vaginal delivery; CS: caesarean section

Pregnancy outcome					p-value
	Underweight n=7 (%)	Normal n=77(%)	Overweight n=77 (%)	Obese n=70 (%)	
Maternal complications					
Yes	2 (28.6)	17 (22.1)	15 (19.5)	21 (30.0)	0.477*
No	5 (71.4)	60 (77.9)	62 (80.5)	49 (70.0)	
Preterm birth					
Yes	1 (14.3)	7 (9.1)	12 (15.6)	11 (15.7)	0.599*
No	6 (85.7)	70 (90.9)	65 (84.4)	59 (84.3)	
Mode of delivery					
CS	4 (57.1)	26 (33.8)	41 (53.2)	44 (63.8)	0.013^#^
SVD	3 (42.9)	51 (66.2)	36 (46.8)	25 (36.2)	
Maternal death					
Yes	1 (14.3)	0 (0.0)	0 (0.0)	2 (2.9)	0.006^#^
No	6 (85.7)	77 (100)	77 (100)	68 (97.1)	
Foetal birth weight					
Low birth weight (<2.5kg)	2 (28.6)	4 (5.2)	3 (3.9)	12 (17.1)	0.001*
Normal (2.5 – 3.9kg)	5 (71.4)	73 (94.8)	65 (84.4)	50 (71.4)	
Macrosomia (≥4.0kg)	0 (0.0)	0 (0.0)	9 (11.7)	8 (11.4)	
Birth asphyxia (APGAR at 1 minute)					
Yes	1 (16.7)	8 (10.4)	6 (7.8)	7 (10.4)	0.050*
No	5 (83.3)	69 (89.6)	71 (92.2)	60 (89.6)	
NNU admission					
Yes	1 (14.3)	5 (6.5)	9 (11.7)	11 (15.7)	0.359*
No	6 (85.7)	72 (93.5)	68 (88.3)	59 (84.3)	
Stillbirth					
Yes	1 (14.3)	0 (0.0)	0 (0.0)	3 (4.3)	0.008^#^
No	6 (85.7)	77 (100)	77 (100)	67 (95.7)	

In Table 4, we present a summary of the association between GWG and pregnancy outcomes. GWG was not associated with any of the outcome measures (p >0.05).

**Table 4:** Association between gestational weight gain and pregnancy outcomes (n = 231) # Fisher's exact test. One pregnant woman with inadequate gestational weight gain did not have her mode of delivery recorded. SVD: Spontaneous vaginal delivery; CS: caesarean section

Pregnancy outcome				p-value
	Inadequate GWG n = 174 (%)	Normal GWG n = 54 (%)	Excessive GWG n = 3 (%)	
	Maternal complications					
Yes	39 (22.4)	15 (27.8)	1 (33.3)	0.668^#^
No	135 (77.6)	39 (72.2)	2 (66.7)	
Preterm birth				
Yes	26 (14.9)	4 (7.4)	1 (33.3)	0.217^#^
No	148 (85.1)	50 (92.6)	2 (66.7)	
Mode of delivery				
SVD	85 (49.1)	29 (53.7)	1 (33.3)	0.908^#^
CS	88 (50.9)	25 (46.3)	2 (66.7)	
Maternal death				
Yes	3 (1.7)	0 (0.0)	0 (0.0)	0.608^#^
No	171 (98.3)	54 (100)	3 (100)	
Foetal birth weight				
<2.5 kg	19 (10.9)	2 (3.7)	0 (0.0)	0.085^#^
2.5 – 3.9 kg	146 (83.9)	44 (81.5)	3 (100)	
≥4.0 kg	9 (5.2)	8 (14.8)	0 (0.0)	
Birth asphyxia (APGAR at 1 minute)				
Yes	16 (9.4)	6 (11.1)	0 (0.0)	0.772^#^
No	154 (90.6)	48 (88.9)	3 (100)	
NNU admission				
Yes	22 (12.6)	4 (7.4)	0 (0.0)	0.468^#^
No	152 (87.4)	50 (92.6)	3 (100)	
Perinatal death				
Yes	4 (2.3)	0 (0.0)	0 (0.0)	0.513^#^
No	170 (97.7)	54 (100)	3 (100)	

**Figure 1:**
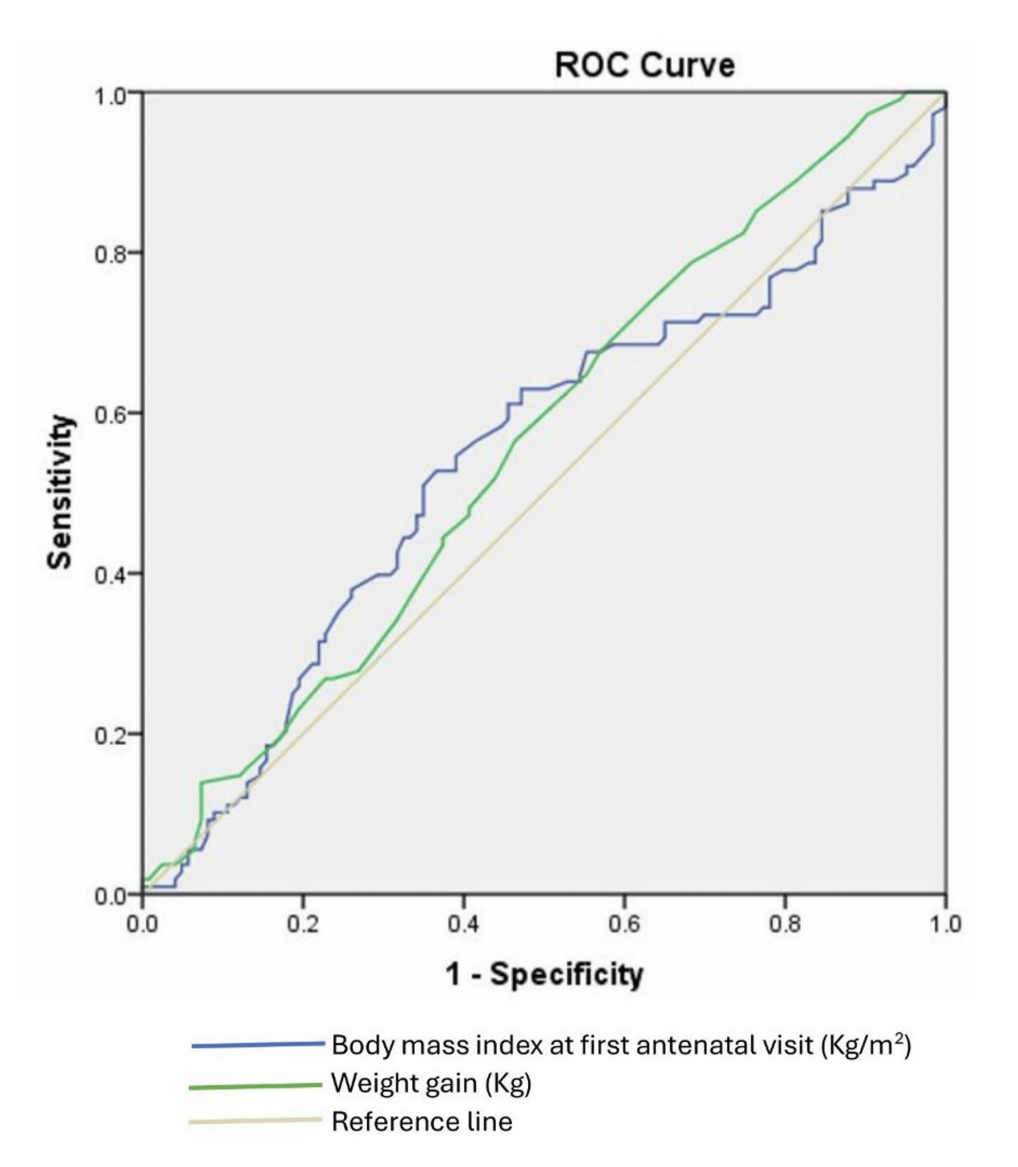
Comparative accuracy of body mass index and maternal weight gain during pregnancy for predicting adverse pregnancy outcomes ROC: Receiver operating characteristic

Figure 1 shows receiver operating characteristic curves comparing the accuracy of BMI and GWG during pregnancy for predicting adverse pregnancy outcomes. Neither BMI nor GWG was found to be a good predictor of a woman having any adverse maternal or foetal outcome. The AUC for BMI as a predictor was poor at 0.547 (95%CI: 0.472 - 0.723) at an optimal cut-off point of 27.95kg/m^2^ and a sensitivity of 52.8% and a specificity of 63.4%. The AUC for GWG as a predictor was 0.561 (95%CI: 0.487 - 0.635) at an optimal cut-off point of 5.75 kg and a sensitivity of 74.1% and a specificity of 36.6%.

## Discussion

This study, which evaluated the association between BMI and GWG and adverse pregnancy outcomes, provides evidence that BMI, but not GWG, is associated with a higher incidence of some adverse maternal and foetal outcomes. It is found that the incidence of stillbirths, caesarean section, and maternal death is higher in pregnant women who are underweight or obese compared to their counterparts with normal BMI. Overweight and obese status are associated with a higher incidence of foetal macrosomia. However, BMI and GWG alone were insufficient in predicting the occurrence of adverse pregnancy outcomes.

The association of BMI with caesarean section is consistent with findings from a previous study in Dhaka, Bangladesh [23]. In addition, the study in Dhaka found a statistically significant association between foetal macrosomia and an overweight/obese mother compared to the normal weight group [23]. This aligns with our study and findings from another study conducted in Chennai, India [24]. The higher incidence of caesarean section in obese women may partly be linked to the higher incidence of foetal macrosomia in them, which can cause cephalopelvic disproportion [25]. However, it is difficult to draw this conclusion because we did not adjust for feotal macrosomia in our analysis. Obese pregnant women have a higher risk of developing medical complications like diabetes, gestational hypertension, or preeclampsia, which increases their risk of having a caesarean section. In addition, it is technically more challenging to monitor the foetus during labour in obese women, especially when intermittent auscultation is used for foetal heart rate monitoring. This might result in a higher likelihood of caesarean section in them, especially in settings with low human resources, and the fear of litigation should sudden foetal death occur. Furthermore, they are more prone to labour dystocia [25].

We found a significant association between stillbirth and underweight and obese pregnant women, which contrasts with the findings of Ali et al. [23], who observed no statistically significant association. However, there was a slightly higher number of stillborn in the overweight/obese group compared to the normal weight group [23]. Our study revealed no significant association between NNU and BMI status, which is consistent with the findings of Dogra et al. [4] and Hemalatha and Shanthini [24]. We observed in our study that there was no significant association between the APGAR score at one minute and BMI status, which is in contrast with the findings of Dogra et al. [4], who observed that a low APGAR score (< 7 at one minute) was significantly higher in the overweight/obese group [4].

In our study, there was no association between GWG and pregnancy outcomes. Similar to our study, Singh et al. [16] did not observe any statistically significant difference in NICU stay between different GWG groups. In contrast to our finding, Singh et al. [16] reported that a significant association between GWG and Apgar score. In their study, they noted that poor APGAR scores of 6 and 7 at one minute were more common among neonates of women with GWG above and below the recommended group, as compared to women with the recommended GWG group.

We observed in this study that BMI and GWG do not have meaningful predictive accuracy for adverse pregnancy outcomes. This is suggestive of the fact that cofounders such as pre-existing medical conditions, socioeconomic status, or access to prenatal care may have played a significant role in determining the pregnancy outcomes.

These findings from our study have huge implications for prenatal care and counselling. We hereby stress that healthcare providers should closely monitor underweight and obese pregnant women during antenatal care for potential complications and also provide targeted interventions to avoid foetomaternal risks. Furthermore, there is a need to emphasise healthy lifestyle choices and weight management preconceptionally and antenatally to improve foetomaternal outcomes.

This study is limited by the fact that it is retrospective in design with a relatively small sample size, especially in the subgroup analysis, which increases the risk of type II error, and as such, it cannot be used with confidence to extrapolate accurately to the general population. In the future, prospective studies with larger sample sizes are recommended to confirm our findings and also explore other factors that might be influencing pregnancy outcomes. In addition, instrument bias is a potential limitation of retrospective studies of this nature. To avert this, we enrolled participants over a short period of three years to increase the likelihood that the same weighing scale would have been used to weigh the pregnant women during the study period. There was limited data to perform multivariable adjustment for confounders like lifestyle, socioeconomic status, and physical inactivity, and a lack of multiple testing correction in the analysis. It would be relevant to take these into consideration in future related studies. 

## Conclusions

Being underweight or obese is associated with a higher incidence of stillbirths, caesarean section, and maternal death in pregnant Nigerian women. In addition, being overweight or obese is associated with a higher incidence of foetal macrosomia. Abnormal BMI does not have any significant association with the incidence of preterm birth, birth asphyxia, and neonatal unit admission. Unlike abnormal BMI, abnormal GWG does not have any adverse effect on maternal and foetal outcomes. However, BMI and GWG alone are not meaningfully predictive of adverse pregnancy outcomes, suggesting that there are likely other factors, such as sociodemographic factors like educational attainment, nutritional deficiencies, lifestyle behaviours and stressor factors, pre-existing medical conditions, and obstetrical issues during pregnancy, which play important roles in predicting adverse pregnancy outcomes.
